# Case Report: Identification of a Heterozygous XPA c.553C>T Mutation Causing Neurological Impairment in a Case of Xeroderma Pigmentosum Complementation Group A

**DOI:** 10.3389/fgene.2021.717361

**Published:** 2021-08-16

**Authors:** Juan Antonio García-Carmona, Matthew J. Yousefzadeh, Fernando Alarcón-Soldevilla, Eva Fages-Caravaca, Tra L. Kieu, Mariah A. Witt, Ángel López-Ávila, Laura J. Niedernhofer, José Antonio Pérez-Vicente

**Affiliations:** ^1^Department of Neurology, Santa Lucia University Hospital, Cartagena, Spain; ^2^Department of Biochemistry, Molecular Biology, and Biophysics, Institute on the Biology of Aging and Metabolism, University of Minnesota, Minneapolis, MN, United States; ^3^Department of Dermatology, Santa María del Rosell University Hospital, Cartagena, Spain; ^4^Unit of Neuromuscular Disorders, Santa Lucia University Hospital, Cartagena, Spain

**Keywords:** xeroderma pigmentosum group A, neurode generation, DNA repair activity, nucleotide excision repair, mutation, rare diseases

## Abstract

We aimed to determine if an adolescent patient presenting with neurological impairment has xeroderma pigmentosum (XP). For this purpose, whole-exome sequencing was performed to assess mutations in *XP* genes. Dermal fibroblasts were established from a skin biopsy and XPA expression determined by immunoblotting. Nucleotide excision repair (NER) capacity was measured by detection of unscheduled DNA synthesis (UDS) in UVC-irradiated patient fibroblasts. Genetic analysis revealed two recessive mutations in *XPA*, one known c.682C>T, p.Arg228Ter, and the other c.553C>T, p.Gln185Ter, only two cases were reported. XPA protein was virtually undetectable in lysates from patient-derived fibroblast. The patient had significantly lower UV-induced UDS (3.03 ± 1.95%, *p* < 0.0001) compared with healthy controls (C5RO = 100 ± 12.2; C1UMN = 118 ± 5.87), indicating significant NER impairment. In conclusion, measurement of NER capacity is beneficial for the diagnosis of XP and in understanding the functional impact of novel mutations in *XP* genes. Our findings highlight the importance of neurologists considering XP in their differential diagnosis when evaluating patients with atypical neurodegeneration.

## Introduction

The detrimental effects of DNA damage are highlighted in patients with genome instability disorders, which can increase the risk of cancer, accelerate aging, and promote early onset neurodegeneration. Xeroderma pigmentosum (XP) is a rare autosomal genome instability disorder caused by a defect in nucleotide excision repair (NER), which is responsible for the removal of bulky DNA adducts that distort the DNA helix. Adducts caused by ultraviolet radiation are a key substrate for NER. Defective NER increases the risk of skin cancer by as much as 10,000-fold, and ~15% of the patients will display progressive neurodegeneration (Digiovanna and Kraemer, 2012; Fu et al., 2016). The estimated prevalence of XP in Europe and the USA is one in 2.3 million persons (Kleijer et al., 2008). Xeroderma pigmentosum complementation group A (XP-A) (OMIM: #278700, Xeroderma pigmentosum I [XP1]) is caused by mutations in the *XPA* gene (9q22.33). The gene product XPA is a key component of NER that helps register the presence of DNA damage and recruits key enzyme to excise the damage from the genome (Mocquet et al., 2008). XPA is required for NER and serves as a scaffold protein and can bind to DNA through its central DNA-binding domain. Many NER and other DNA repair factors like DDB2, ERCC1, RPA32/70, and TFIIH bind to XPA in order to complete the NER process. For instance, XPA forms the pre-incision complex with TFIIH, RPA, and XPC-HR23B and then is responsible for recruitment of the structure-specific endonuclease ERCC1-XPF. Thus, loss of XPA prevents the excision of damaged DNA (Borszekova Pulzova et al., 2020).

Mutations in *XPA* often severely impair NER and are quite common particularly in Japan. XP-A patients often have extreme photosensitivity and frequent skin cancers in sun-exposed areas of the skin. In addition, 15% of XP-A patients have neurological involvement beginning in the third decade of life (Bowden et al., 2015). XP neurologic disease is driven by primary neurodegeneration and is clinically characterized by a progressive loss of cognitive and motor skills, hearing loss, microcephaly, and/or peripheral neuropathy (Lai et al., 2013).

Here, we report a case of an adolescent Caucasian female suffering from progressive cognitive impairment, deafness, neuropathy, muscle atrophy and ataxia, freckle-like skin lesions, and some verrucous epidermal nevus but no skin cancer. Whole-exome sequencing revealed two heterozygous mutations in *XPA one* of indeterminant impact. Analysis of patient cells revealed low expression of XPA and impaired NER, confirming the diagnosis of XP. In clinical practice, XP is well known as causing skin lesions, but patients with neurological impairment, as first symptom, are not suspected to suffer XP. Therefore, an early genetic diagnosis might prevent complications associated with unprotected exposure to sunlight. Moreover, the analysis of DNA repair capacity has prognostic value because it is related with disease severity. Altogether, these findings highlight the importance of neurologists considering XP in the differential diagnosis when evaluating patients with atypical neurodegeneration.

## Methods

### Study Protocol, Ethics Approval, and Patient's Consents

Ethics approval was granted by the Santa Lucia Hospital Clinical Ethics Committee. Written informed consent was obtained from patient's mother to carry out the study as well as to use representative images and photographs with clinical and teaching interest.

The patient had completed extensive clinical evaluations in the last 5 years, including investigations for toxic, metabolic, infectious, autoimmune, and inflammatory processes and had undergone genetic testing for common hereditary neurodegenerative disorders. Additional clinical details are summarized in the Table 1.

**Table 1:** XP-A patient's molecular, clinical and neurological findings.

***XPA* Mutations**	**c.553C>T, p.Gln185Ter (exon 4) c.682C>T, p.Arg228Ter (exon 6)**
Age	
First medical examination	9 years old
Current	14 years old
Ethnicity	Caucasian, Mediterranean European
History of sun-burning on minimal exposure	Yes
Freckle-like skin lesions	Mild
Skin cancer	No
Symptom prompting first neurological examination	Cognitive decline at 9 years of age
Sequence of neurological symptoms	Cognitive decline, hearing loss, ataxia, neuropathy
Height (cm); weight (Kg)	157; 45Kg; BMI = 18.3
Developmental milestones	Normal
Education	Secondary School
Speech	Ataxic dysarthria
Hearing	Neurosensory hearing loss
Tendon reflexes, plantar response	+/++++; indifferent
Activities of daily living	Needs some assistance
Urinary incontinence	No
Other neoplasm history	No
Optic disc, retina	Normal
Brain imaging	Normal
DNA repair (UDS)	3.0 ± 1.95% of control

*BMI, body mass index; UD, unscheduled DNA synthesis*.

### Patient Sequencing

DNA was extracted from peripheral blood, and whole-exome sequencing was performed using the QGenExWES kit (QGenomics, Barcelona; Spain) on a NovaSeq 6000 (Novogene, UK) at the Genetic Laboratory (Virgen Arrixaca University Hospital, Murcia; Spain). In accordance with the genome analysis best practices guidelines, we mapped sequence reads to the human reference genome using the Burrows-Wheeler Aligner (v.0.8), removed duplicate reads (Picard v.1.11), and identified SNPs and Indels (SAMtools v.1.0). Pathogenic and likely pathogenic variants considered to be of clinical significance were confirmed by Sanger sequencing, and, when available, parents were also tested by Sanger sequencing to determine segregation.

### Generation of Dermal Fibroblast Lines

Skin punch biopsies (5 mm) were used to create dermal fibroblast primary cells from the patient with mutations in *XPA* (XP1UMN) and an unrelated control individual (C1UMN). Initially, biopsies were cultured in α-MEM with EGF (10 μg/ml), bFGF (10 μg/ml), 10% fetal bovine serum, and 1% PenStrep (Thermo Fisher, Waltham, MA, USA) in an incubator at 37°C in a 5% CO_2_ at ambient oxygen concentration. After initial passage of dermal fibroblast lines, they were then cultured in DMEM + Glutamax (Thermo Fisher) with 10% fetal bovine serum and 1% PenStrep.

### Immunoblotting

Whole cell extracts were prepared from cell pellets using RIPA buffer before addition of 2 × Laemmli buffer and heat denaturation. Lysates were electrophoresed on 4%−15% gradient gels before being transferred to nitrocellulose membrane. Immunoblots were blocked in 10% milk TBST before being probed with anti-XPA (GeneTex, Irvine, CA, USA, catalog #GTX103168, 1:500) and anti-α-Tubulin (Abcam, Cambridge, MA, USA, ab4074, 1:1,000) primary antibodies and the corresponding anti-rabbit HRP secondary antibody (Sigma, St. Louis, MO, USA, A0545, 1:5,000). Bands were visualized using ECL chemiluminescence on an iBright (Thermo-Fisher) imaging system.

### Measurement of Unscheduled DNA Synthesis

Unscheduled DNA synthesis (UDS) was measured in patient fibroblasts as described (Mori et al., 2018; Shanbhag et al., 2018). Dermal fibroblast lines C5RO (control), C1UMN, XP51RO (XFE progeroid syndrome), and XP1UMN (patient) were irradiated with 24 J/m^2^ UV-C or mock-irradiated. XP51RO cells were from a patient with a homozygous p.Arg153Pro *ERCC4* mutation associated with XFE progeroid syndrome and severely reduced UDS (<3%) (Niedernhofer et al., 2006). Following irradiation, the cells were incubated in the presence of the thymidine analog EdU (10 μM) for 2.5 h to allow DNA repair. After permeabilization by saponin, Alexa Fluor 647 was conjugated to EdU using Click-It chemistry. Cells were then fixed in 4% formaldehyde and stained with DAPI to determine the cell cycle profile. Median Alexa Fluor 647 fluorescence intensity in G1 cells was measured by flow cytometry. UDS was measured in duplicate for each cell line in three independent experiments. All cells were grown in DMEM + Glutamax with 10% fetal bovine serum and 1% PenStrep in an incubator at 37°C in 5% CO_2_ at ambient oxygen concentration. All flow cytometric data was analyzed using FlowJo 10.0 (FlowJo, Ashland, OR, USA).

## Results

A 14-year-old Caucasian female, of Mediterranean European descent, with a history of skin photosensitivity but no skin cancer received medical attention at the age of 9 years for progressive cognitive decline and hearing loss. Her parents were both European Caucasian, and no history of consanguinity was reported. Previous newborn metabolic and hearing screening tests were normal. Speech-language and psychomotor development were normal, including walking with 1 year and sphincter control with 2.5 years.

**Figure 1:**
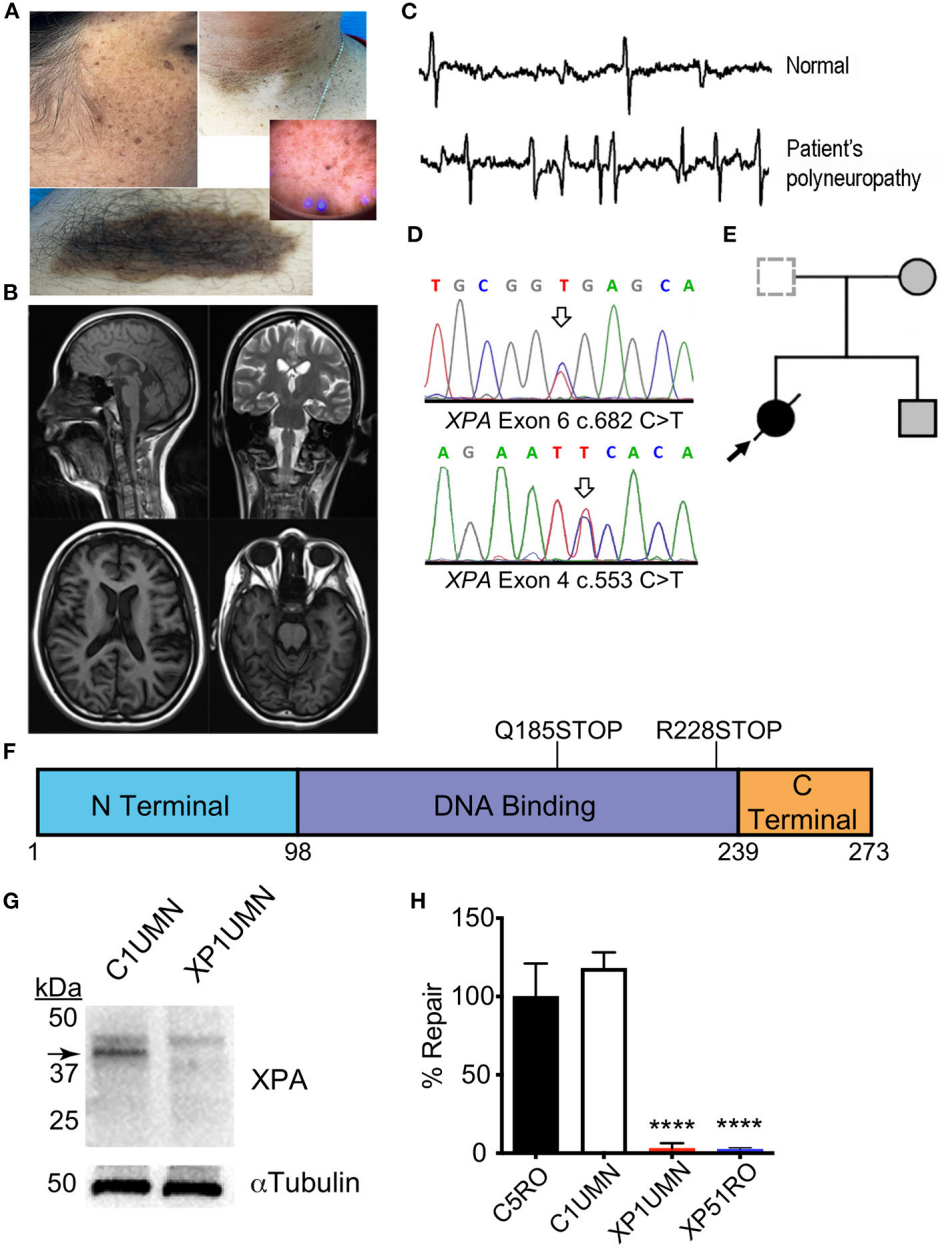
Clinical and functional impact of a known and novel mutation in *XPA*. **(A)** Face and arm photographs demonstrating prominent skin freckling or verrucous epidermal nevi. **(B)** MRI images showing no signs of pathology. **(C)** Representative image of EMG/nerve conduction tests indicating diffuse axonal sensorimotor polyneuropathy. **(D)** Sanger chromatogram demonstrating relevant mutations in both *XPA* alleles from the patient (arrows). **(E)** Nuclear family pedigree. **(F)** Location of *XPA* mutations. Schematic diagram of XPA protein with the functional domains of XPA. Each mutation gives rise to a premature stop codon. The corresponding amino acid numbers are shown below. **(G)** Mutations cause a loss of XPA in patient fibroblasts. Immunoblotting for XPA in patient dermal fibroblasts from suspected XP-A patient (XP1UMN) and an unrelated control (C1UMN). α-Tubulin was used as a loading control. Arrow denotes the specific band corresponding to XPA. **(H)** Measurement of UV-induced UDS in patient fibroblasts. Unscheduled DNA synthesis was measured in fibroblast lines, and the relative NER capacity is reported. NER capacity was normalized to those obtained for NER-proficient (C5RO) cells (set at 100%), and NER-deficient (XP51RO) control fibroblasts were used as a negative control. UDS was measured in duplicate for each cell line in three independent experiments. Values represent mean ± SD, ****p < 0.0001 by one-way ANOVA with Tukey's test.

Skin examination showed a phototype 4 on the Fitzpatrick scale (Fitzpatrick, 1988) and skin lesions (Figure 1A) including buttock hemangioma and common warts were present, although none was cancerous. Neurological examination showed progressive mild intellectual impairment, deafness, cavus foot, hypoesthesia in glove and sock, atrophy in legs, and ataxia (Table 1). Brain MRI (Figure 1B) showed no alterations while EMG and nerve conduction studies (Figure 1C) indicated diffuse axonal sensorimotor polyneuropathy. Two years prior the present study, common diseases causing neurodegeneration in the childhood, such as Charcot-Marie-Tooth syndrome and porphyrias were discarded by using genetic tests and karyotyping. Nonetheless, suspecting XP, genetic testing was ordered.

Following whole-exome sequencing, variants were prioritized based on known disease association and population frequency as well as clinical skin phenotype. Our patient had compound heterozygous mutations in *XPA* (Figure 1D). The patient carried one known mutation associated with *XPA*; NM_000380.3: c.682C>T, p.Arg228Ter (allelic frequency < 0.03%) and one unknown mutation c.553C>T, p.Gln185Ter. Segregation analysis of the family confirmed compound heterozygous allele pairs (Figure 1E). The known mutation causes a stop codon in the C-terminal domain while the novel mutation creates a stop codon in the DNA-binding domain of XPA (Figure 1F). This combination of mutations dramatically reduced XPA protein abundance in dermal fibroblasts from the patient (XP1UMN) compared with those from an unrelated control (C1UMN) (Figure 1G). UV-induced unscheduled DNA synthesis was measured on the patient fibroblasts. UV-induced UDS is a direct measure of NER capacity and serves as gold standard for investigating possible NER deficiency, which is characteristic for the diagnosis of XP complementation group A (Yamaguchi et al., 1990; Lehmann et al., 2011). NER-proficient (C5RO) and NER-deficient (XP51RO) fibroblast lines (Niedernhofer et al., 2006; Mori et al., 2018; Shanbhag et al., 2018) were used as controls. The patient fibroblasts (XP1UMN) had a significant reduction in UDS (3.03% ± 1.95%, *p* < 0.0001) compared with controls (C5RO = 100 ± 12.2; C1UMN = 118 ± 5.87), indicating significant NER impairment (Figure 1H). Thus, the lack of XPA protein, reduced UV-induced UDS, and the clinical features are consistent with a diagnosis of XP.

## Discussion

We found a mutation (c.682C>T; p.Arg228Ter) present in exon six in one of the alleles of *XPA*. This mutation have been described in nine Moroccan patients suffering severe skin lesions while neurological disorders were also observed in three aged (>30 years old) patients (Kindil et al., 2017). This mutation causes a premature stop signal and results in a protein with a truncation in the C-terminal region of XPA where TFIIH, another component of the NER machinery, binds. XPA has a predicted molecular weight of 31 kDa but migrates to ~40 kDa in SDS-PAGE gels (Asahina et al., 1994). Previous immunoblotting of XP39OS cells, which have pR228Ter mutation in both alleles, showed XPA-specific bands of 33 and 35 kDa (Tanaka, 1993). Further optical densitometry analysis showed an 80% reduction in total XPA abundance compared with control cells. In the other allele of *XPA*, a c.553C>T mutation in exon 4 (Gln185Ter) was shown. This mutation has been only reported as homozygous in two Egyptian patients with parents' consanguinity. This two patients suffered severe and progressive mental retardation and neurodegeneration from childhood (8 years old) (Amr et al., 2014). Altogether, we suggest this mutation could be underlying XP-A with severe neurological involvement at early age onset. Because this variant produces a nonsense mutation in exon 4, it is expected to cause a truncation in the DNA-binding domain of XPA (Satokata et al., 1992a,b). Immunoblotting of XP1UMN fibroblasts showed a dramatic reduction in XPA and no lower molecular weight bands (Figure 1G). This could be due to the following: (1) the anti-XPA antibody used for immunoblotting was raised against the central region of XPA and one or both of the mutation sites could be contained in the epitope; (2) the antibody binds to XPA past the mutation sites and thus lack the ability to detect the truncated forms of XPA; or (3) both truncated forms of XPA could be unstable. Thus, the patient does not produce full-length functional XPA protein. Loss of XPA impedes NER (Scharer, 2013), consistent with the diagnosis of XP with neurological impairment.

NER defects may be associated with profound neurodegeneration in XP (Satokata et al., 1992b). The underlying mechanism is not yet fully understood. The presumption is that neurodegeneration is caused by failure to repair endogenous DNA lesions caused, for example, by oxidative stress. Cyclopurine adducts, for example, are oxidative DNA lesions that are substrates for NER and are detected in the brain and other tissues of mammals (Wang et al., 2012). Nonetheless, further studies are needed to truly establish the role of NER in neurodegenerative diseases. As previously suggested, mutations in exons 3, 4, and 5 of XPA may be related to neurological abnormalities (Rabie et al., 2021). It will be necessary to improve the understanding of the etiology of the neurological problems in order to develop treatments to relieve or prevent neurodegeneration in XP patients.

In conclusion, we report for the first time the mutation c.553C>T in *XPA* in a European Caucasian patient with severe NER impairment. We suggest that c.553C>T mutation might be underlying XP-A with severe neurological involvement. Moreover, NER analysis is useful as disease prognostic value and may help to assess the severity of the XP mutations. Finally, the diagnosis of XP, and particularly XP complementation group A, should be considered in patients with unexplained neurodegeneration especially when accompanied by a history of photosensitivity or skin malignancies.

## Data Availability Statement

The datasets for this article are not publicly available due to concerns regarding participant/patient anonymity. Requests to access the datasets should be directed to the corresponding author.

## Ethics Statement

The studies involving human participants were reviewed and approved by Santa Lucia Hospital's Ethics Research Committee. Written informed consent to participate in this study was provided by the participants' legal guardian/next of kin. Written informed consent was obtained from the individual(s), and minor(s)' legal guardian/next of kin, for the publication of any potentially identifiable images or data included in this article.

## Author Contributions

JG-C wrote the initial draft of the manuscript, study design, sample logistics, and interpretation of data. MY and TK measured XPA protein in fibroblasts. FA-S and ÁL-Á clinical consult and procured skin samples. EF-C and JP-V clinical consult. MY and MW measured UV-induced nucleotide excision repair in human fibroblasts and analyzed data. MY and LN helped with data interpretation and manuscript writing.

## Conflict of Interest

JG-C serves or has served on the Editorial Board of *BMC Neuroscience*. MY has received research support from the American Federation on Aging Research. LN has received research support from the NIH/NIEHS, NIH/NIA, and Glenn Award for Aging Research and is the co-founder of NRTK Biosciences, a start-up biotechnology company developing senolytic drugs. The remaining authors declare that the research was conducted in the absence of any commercial or financial relationships that could be construed as a potential conflict of interest.

## Publisher's Note

All claims expressed in this article are solely those of the authors and do not necessarily represent those of their affiliated organizations, or those of the publisher, the editors and the reviewers. Any product that may be evaluated in this article, or claim that may be made by its manufacturer, is not guaranteed or endorsed by the publisher.
